# Natural, small molecule aliphatics (cholesterol and hexadecyl palmitate) as dielectrics for low-voltage organic field effect transistors

**DOI:** 10.1039/d5ma00730e

**Published:** 2026-01-27

**Authors:** Cristian Vlad Irimia, Corina Schimanofsky, Boyuan Ban, Cigdem Yumusak, Martin Ciganek, Petr Sedlacek, Jozef Krajcovic, Rosarita D’Orsi, Alessandra Operamolla, Andreas Petritz, Katharina Matura, Barbara Stadlober, Yasin Kanbur, Yolanda Salinas, Oliver Brüggemann, Christian Teichert, Niyazi Serdar Sariciftci, Mihai Irimia-Vladu

**Affiliations:** a Linz Institute for Organic Solar Cells (LIOS), Institute of Physical Chemistry, Johannes Kepler University Linz Altenberger Str. 69 4040 Linz Austria mihai.irimia-vladu@jku.at; b Chair of Physics, Department of Physics, Mechanics, and Electrical Engineering, Montanuniversität Leoben Franz Josef Str. 18 8700 Leoben Austria; c Institute of Solid-State Physics, Hefei Institute of Physical Science, Key Lab of Photovoltaic and Energy Conservation Materials Hefei China; d Brno University of Technology, Faculty of Chemistry, Purkyňova 464/118 612 00 Brno Czechia; e Department of Chemistry and Industrial Chemistry, University of Pisa, via Giuseppe Moruzzi 13 54126 Pisa Italy; f joanneum Research Materials-Institute for Surface Technologies and Photonics, Franz-Pichler Strasse 30 8160 Weiz Austria; g Department of Chemistry, Karabük University, Baliklarkayasi Mevkii Karabük 78050 Turkey; h Institute of Polymer Chemistry, Johannes Kepler University Linz Altenberger Str. 69 Linz 4040 Austria; i IMC Krems University of Applied Sciences, Institute of Applied Chemistry, Piaristengasse 1 3500 Krems Austria

## Abstract

In this study, we show that hexadecyl palmitate and cholesterol, two naturally occurring small molecule aliphatics, are suitable dielectrics for organic field effect transistors (OFETs). We provide a comprehensive description of their material characteristics, processability, and film-forming capabilities, as well as surface characterization and dielectric analysis. We finally employ them for the fabrication of organic field effect transistors, employing two traditional organic semiconductors, pentacene and fullerene, C_60_. We demonstrate that most OFETs can function with operating voltage windows as low as 1 V, and driving voltages as low as 10 mV, when these materials solubilized in chloroform, are fabricated utilizing blade coating technique.

## Abstract 2

Two, naturally occurring small molecules, cholesterol and hexadecyl palmitate are demonstrated to be excellent dielectric materials for organic field effect transistors operating at low voltage in the frame of sustainable electronics development.

## Graphical abstract

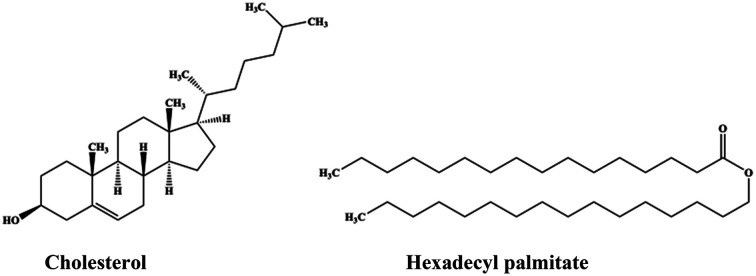

## 1. Introduction

The increasing demand for electronics of the consumers market, and the strategy of the producers to offer to customers only disposable products, without the possibility to upgrade their technological obsolescence, puts pressure on the availability of critical materials, and causes a sharp rise in electrical and electronic equipment waste.^1^ Moreover, the current fabrication of high performance electronics, not only engages complex, and energy inefficient methods,^2,3^ but also employs hazardous materials like halogenated compounds, heavy metals and even radioactive substances (*i.e.*, americium, that is widely used for ionization type smoke detectors). These events raise serious issues related to society, environment, health, and even geopolitics.^4^ While high-end electronics are fabricated predominantly with the aid of inorganic materials, scientists discovered in the past cheaper alternatives, in the name of organic counterparts, that contribute to the fabrication of sustainable, biocompatible and even biodegradable devices.^5^ The organic electronics field is based mostly on solution-processable organic components that can even be derived from natural sources or created using the principles of green chemistry.^6–10^ It was anticipated that solution-processable (printable) organic materials would result in low embodied-energy electronics, because their processing occurred under ambient conditions, meaning that neither high vacuum nor high temperature were necessary.^11–17^ Therefore, organic electronic component materials appear to be ideal for applications that interface the electronics and sensor world with living systems in order to develop disposable diagnostic and drug-delivery technologies, because of their inherent softness and flexibility, which match the elastic modulus of living cells.^6,7,18–36^ Although the field of synthetic chemistry contributed to tremendous recent advancement in performance of organic materials (substrates, dielectrics, semiconductors, and even conductors), many other challenges on the side of materials and their performance remain to be achieved, in order to lessen the environmental impact of e-waste.^37–48^ Mainly due to the quick expansion of applications for disposable electronic and sensor devices,^49–51^ there is nowadays a greater need not only for the development of sustainable waste management solutions but also for the identification of green and biodegradable materials.^52–59^ In one such thrust, scientists searched for reducing the volume of the materials employed in the fabrication process of electronics.^60–63^ Employing materials originating directly from nature represented another viable drive, meant to address the environmental impact of electronics.^18,30,55,56,64–74^ Among the variety of scientific reports, the organic field effect transistor device proved itself to be a suitable platform for testing new materials and devices at laboratory level, despite its inherent limitation compared to its inorganic based counterparts, originating primarily from the lower purity level of component materials, their inherent disorder, and weak intermolecular bonds.^75–80^ Although traditional FETs typically use inorganic materials like silicon dioxide, silicon dioxide (SiO_2_), aluminum oxide (Al_2_O_3_),^81–84^ and other metal oxides, like those of titanium, hafnium, tungsten, and tantalum for the insulating layer, OFETs typically employ either a fully organic dielectric layer or a combination of thin inorganic dielectric capped by an organic layer.^85^ Although the performance of inorganic based FETs is unequivocally higher than that of their organic counterparts, the scientific community is becoming more interested in developing OFETs using organic materials that are non-toxic, renewable, environmentally benign (even biodegradable), and inexpensive, allowing for simple processing at low temperatures.^86,87^

Materials known as dielectrics react to electromagnetic waves, especially those in the optical range, and allow an electrical field to exist for extended periods of time. Because of this, electromagnetic energy builds up, disperses, slows down, is absorbed, and can be changed in dielectrics. Dielectrics' primary feature is electrical polarization, and their electrical conductivity is frequently low. For many electronic applications, dielectrics are essential: in field effect transistors (FETs), through their polarization, the dielectrics provide the capacitance effect, *i.e.*, the necessary charges for the modulation of the conductivity of the semiconductor material at the channel region. They are essential in everything from power systems to sophisticated microchips; insulation (wires, cables); energy storage (capacitors); signal filtering and improving semiconductor performance; insulating layer in capacitors preventing current flow; guiding light in optical fibers; and enabling faster electronics by reducing crosstalk because of their capacity to store charge and withstand high voltages.

The two small molecule dielectric materials reported here fall within the top echelon of the group of low dielectric constant organic dielectric materials (*i.e.*, also known as low-k dielectrics, having their dielectric constant in the range of 3.9 or lower),^88^ like for example cross-linked poly-4-vinylphenol (PVP),^89^ polyvinylidenedifluoride (PVDF),^29,90^ poly(methyl methacrylate) (PMMA),^91–94^ polystyrene (PS),^92^ amorphous fluoropolymer (CYTOP),^95^ commercial SU8 photoresist,^96^ or 1,7-diazaperylene.^97^ However, they seem to fit also into the lower tier of materials in the high-k dielectrics group (*i.e.*, organic insulators with dielectric constants higher than 4.0),^98^*e.g.*, poly(vinyl alcohol) (PVA),^99^ poly(vinyl phenyl) (PVP)^100^ poly(vinylidene fluoride-trifluoroethylene-chlorofloroethylene) (P(VDF-TrFE-CFE)),^101^ and polyacrilonitrile (PAN).^102^ However, with notable exceptions^95,100,101^ nearly all the contributions mentioned above report OFETs comprising respective dielectrics having high operating voltages, in some instances as high as 30 V or higher.^89,90,93,94^ One of our main objectives was to provide the scientific community materials processible in thin films that afford fabrication of organic field effect transistor devices operating at voltages as low as 1 V, and ideally in millivolts regime. In addition, the two small molecule dielectrics presented here represent an addition to the portfolio of natural dielectrics reported in the literature, like for example natural waxes, plant resins, animal resin shellac, silk, purine nucleobases, alkaloids, various sugars, lignin and celluloses.^57,65,68,70,71,73,74,103–114^

Within the pool of natural materials reported in the literature in the past years for organic electronics applications, the two small molecules investigated in this study stand out in terms of their inherent biocompatibility, since they are found in large amount in many living organisms or animals on Earth. The article does not intend to compare directly the two small molecules, but provide them as alternative to the abundant data of literature comprising natural dielectric materials in the context of the larger pool of literature comprising synthetic organic small molecules and polymers dielectric materials.

**Scheme 1:**
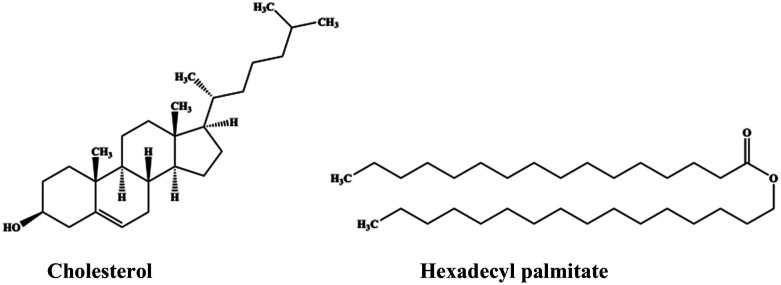
Chemical formulas of cholesterol and hexadecyl palmitate (HDP).

Cholesterol is a sterol found in all of vertebrates on Earth other than fish. It is part of the steroid family of substances and is biosynthesized by animal cells and distributed in several body tissues, especially the brain and spinal cord.^115–117^ It is an essential structural and signaling component of animal cell membranes that is found also in the animal fats and oils. Cholesterol is considered to be a fatty substance, and while its essential presence in the cell membranes is unambiguously demonstrated, its amount has to be carefully managed in order to safeguard the occurrence of cardio-vascular diseases.^118^ In its pure state, cholesterol appears as a white, crystalline substance that is odorless and tasteless. Cholesterol is a cholestanoid, *i.e.*, a steroid substance based on a cholestane skeleton with a 3β-hydroxy group and a double bond at the 5,6-position, having the IUPAC name (3β)-cholest-5-en-3-ol (see Scheme 1). It melts upon heating at ∼ 150 °C, and decomposes when reaching the boiling temperature of ∼360 °C. It is slightly soluble in ethanol at room temperature but has a higher solubility in a variety of other solvents, including chloroform, pyridine, acetone, dioxane, ethyl acetate, benzene, petroleum, ether, oils and fats (see ref. 119 and references therein).

Hexadecyl palmitate (HDP), also referred elsewhere as cetyl palmitate, is an organic compound generally used in the cosmetic industry as a thickener, emulsifier, and moisturizer agent for skin conditioning. In its pure form it is a white-solid, waxy-like substance that occurs in nature as the primary constituent of both stony corals^120^ and spermaceti wax, *i.e.*, the once highly prized substance found and extracted from the skull of sperm whales, *Physeter macrocephalus*. Apart from its application in cosmetics, hexadecyl palmitate finds applications in pharmaceutical industry,^121^ and in food industry as lubricant (anti-sticking agent) for the food packaging.^122^ In addition, it was reported that hexadecyl palmitate's lubricant properties recommend it for the production of stainless-steel *via* cold rolling technique.^123^ Hexadecyl palmitate, is the ester derived from hexadecanoic acid and 1-hexadecanol, with the IUPAC name of hexadecyl hexadecanoate (see Scheme 1).

In this report, we procured the two small molecules from the chemical suppliers, performed an assessment of their inner composition, their processability and film forming capabilities, as well as their dielectric and materials properties, and finally implemented them as dielectric layers in organic field effect transistors operating at low applied voltage.

## 2. Experimental

Cholesterol was purchased from Sigma, product No. 2673, and used without any further purification. Hexadecyl palmitate was purchased from TCI, product No. P1077, and was also used in this study without any further purification. Both materials were solubilized in chloroform in a concentration of 2.5 mg mL^−1^. Although cholesterol is soluble in a variety of other solvents (many of them being coined as “green”, like ethanol, acetone, or methanol), hexadecyl palmitate is more problematic with this respect. We decided to use a unique solvent for both materials in order to account for a better comparison between their dielectric properties and performance in OFETs.

### Elemental analysis

An Elementar Vario Micro Cube analyzer was used to conduct elemental analysis studies. The difference in the contents of carbon, hydrogen, sulphur, and nitrogen was used to determine the oxygen concentration of each sample. Every determination was made twice. In every case, the standard deviation was less than 0.2 atomic %. Five milligram sample of each material were used for the analyses. The dynamic detection ranges of work in CHNS, CNS mode were: C 0.087–7 mg (absolute); H 0.010–1 mg (absolute); N 0.034–10 mg (absolute); S 0.039–2 mg (absolute). The precision of the instrument was less than or equal to 0.1% of the absolute values. By simultaneously determining 2 mg of acetanilide or sulphanilamide (analytical standards) in CHN or CHNS mode, the instrument was periodically calibrated for each operating mode, for each measured element, over the whole measurement range. Additionally, in order to adjust the calibration based on the atmospheric conditions (temperature, pressure) at the time of analysis, the “daily factor” was calculated using standard samples (acetanilide or sulphanilamide) for each analysis session.

### Differential scanning calorimetry (DSC) analysis

A temperature-modulated calorimeter (DSC Q2000, TA Instruments, New Castle, DE, USA) with an RCS90 cooling accessory was used to perform differential scanning calorimetry (DSC). Under a dynamic nitrogen environment, all of the experiments were conducted in TzeroTM aluminum pans that were hermetically sealed (TA Instruments, Lukens, DE, USA). The phase transitions of the samples under analysis were examined using temperature-modulated DSC in the following manner: around 5 µg of a sample was first equilibrated at 200 °C and maintained isothermally at this temperature for 5 minutes. After that, the sample was cooled to 10 °C at a rate of 2 °C per minute. The sample was heated to 100 °C once more using the same temperature modulation as the first heating step, and a heating rate of 2 °C min^−1^ following another equilibration step (5 min at 10 °C). The TA Universal Analysis 2000 program (TA Instruments, Lukens, DE, USA) was used to evaluate the thermograms.

### Thermogravimetric analysis (TGA)

A TGA/PerkinElmer Q5000 was used to perform thermogravimetric analyses on the samples under investigation. The measurements were conducted in the thermal range of 70 °C to 500 °C with a heating rate of 10 °C min^−1^ under nitrogen atmosphere (25 mL min^−1^). The samples were weighed in platinum pans (∼5–27 mg, depending on the sample). Identical experimental setup and protocols were used to measure each small molecule aliphatic.

### Fourier transform infrared (FTIR) spectroscopy

The iS50 FTIR spectrometer (Thermo Scientific, Waltham, MA, USA) was used to record the Fourier transform infrared (FTIR) spectra of the small molecule aliphatics at room temperature (in an air-conditioned environment) using the integrated single-reflection diamond attenuated total reflectance (ATR) crystal. Every ATR spectrum was gathered using an average of 32 scans with a resolution of 4 cm^−1^ (data spacing 0.5 cm^−1^) in the typical mid-infrared spectral range (4000–400 cm^−1^).

### Contact angle measurements

The measurements were performed on a KRÜSS DSA 100 Contact Angle Measuring System, able to provide information about the surface energy of the film, in addition to the contact angle value. The employed liquids were diiodomethane (a very nonpolar liquid with a total energy, *γ* = 50.8 mN m^−1^, separable into a polar component, *γ*^P^ = 0 mN m^−1^, and a dispersive component, *γ*^D^ = 50.8 mN m^−1^); and ultrapure water (a very polar liquid with a total surface tension *γ* = 72.8 mN m^−1^ separable in a polar component *γ*^P^ = 51 mN m^−1^ and a dispersive component *γ*^D^ = 21.8 mN m^−1^). Five droplets of water and diiodomethane, respectively, were used to compute the surface energy for each small molecule aliphatic. The mean values from five droplets, together with their standard derivations, are reported here.

### Atomic force microscopy (AFM) and amplitude modulated Kelvin probe force microscope (AM-KPFM) characterization

Small molecule aliphatic films have been deposited on aluminum coated glass substrates *via* doctor-blading, of a typical thickness of ∼130–150 nm. They were subjected to measurements using atomic force microscopy (AFM) and amplitude modulated Kelvin probe force microscopy (AM-KPFM) by an Asylum Research MFP-3D AFM system. ASYELEC-01-R2 probes (Ti/Ir coating on both the reflective and tip sides) with a tip radius of 25 ± 10 nm, a resonance frequency of 75 kHz, and a spring constant of 2.8 N m^−1^ were used for the AM-KPFM measurements. In the AM-KPFM setup, heights and contact potential differences (CPD) were measured using a two-pass mode. Small molecule aliphatic films were grounded while scanning at a speed of 5 µm s^−1^, then the probe was raised by 10 nm during the second scanning. The averages with standard deviations for the root mean square (RMS) values of topographical roughness and CPD fluctuations were calculated for five randomly selected 20 × 20 µm^2^ areas from each sample. Open-source Gwyddion v2.62 software was used to evaluate topographical and CPD images. Topography images were subjected to first-order line filtering and base plane leveling, whereas CPD images were subjected to zero-order line filtering only.

### Small molecule aliphatics processing in thin films and OFETs fabrication

Before being deposited as thin films, both small molecule aliphatics were filtered through a hydrophobic membrane after being dissolved in chloroform at a concentration of 2.5 mg mL^−1^. We considered drop casting, spin coating, and blade coating (also known as doctor blading elsewhere) as deposition techniques. However, only the latter procedure yielded films of acceptable quality because drop casting and spin coating processes could not regulate the very fast evaporation of the solvent and the subsequent nucleation of the small molecule aliphatics particles. The glass slides were coated using a COATMASTER 509 MC machine. The ideal operating parameters for the blade coating device were: a concentration of 2.5 mg mL^−1^ of small molecule aliphatics in the carrier solvent, a blade height of 0.6 mm, a processing speed of 2.5 mm s^−1^, and an injection volume of 25 µL of precursor material on the back of the moving blade.

For the impedance measurements, the small molecule aliphatics were processed on top of 50 nm thick aluminum electrodes on glass substrates. The metal–insulator–metal (MIM) structure was terminated by the deposition of 50 nm top aluminum electrodes. Both top and bottom aluminum electrodes were deposited *via* physical vapor deposition on an Edwards AUTO 306 Vacuum Coater at a deposition speed of 1 Å s^−1^. We fabricated 4 slides for each investigated material, containing 4 MIM devices on each slide, in a similar way described in our previous work.^70^

This study used an OFET structure on glass substrates with a staggered bottom gate-top contact layout, with the OFET schematic shown in a distinct figure in the text of the manuscript. Four transistors were present on each glass slide; however, they were all connected by a 50 nm thick aluminum gate electrode and doctor-bladed aliphatic small molecule. Using a specialized organic evaporator, a Vaksis Research and Development Evaporator, the two semiconductors used, fullerene, C_60_ as an n-type semiconductor, and pentacene, as a p-type semiconductor, were applied to individual patches, each one being 60 nm thick, on top of the blade-coated small molecule aliphatic. In a vacuum of 1 × 10^−6^ mbar or less, the semiconductor evaporation was carried out at a rate of about 0.1 Å s^−1^. A pair of top electrodes, gold for pentacene, and aluminum for fullerene (C_60_), were used to terminate the structure in an Edwards AUTO 306 Vacuum Coater. The deposition speed was approximately 0.1 to 0.2 Å s^−1^ for the first 5 nm of the deposited layer and approximately 1 Å s^−1^ for the remaining 55 nm of the 60 nm total thickness. A unique pair of source and drain (S–D) electrodes was assigned to each patch of organic semiconductor. The OFET channel dimensions were: length, *L* = 25 µm (*i.e.*, the distance between the source and drain electrodes), and width, *W* = 2 mm (*i.e.*, in reality the width of the gate electrode). An Agilent Technologies A1500B Semiconductor Device Analyzer probe station, mounted in a glove box under nitrogen, was used to measure the OFETs.

## 3. Results and discussion

Table 1 presents the results of the two compounds' elemental analysis examination in comparison to the theoretical values. The values of the hydrogen content obtained are consistent with the standard deviation error (0.2 atomic %) derived from the number of measurements (at least two). The discrepancy for carbon is more than anticipated based on the deviation standard, yet it has no discernible impact. Actually, taking into account the molecular weight, the carbon to hydrogen minimum ratio in the formula derived from elemental analysis validates the relative ratios of C : H 1 : 2 for hexadecyl palmitate and C : H 1 : 1.7 for cholesterol. The amount of carbon causes the oxygen value to diverge from the others, however in this instance, the ratio of hydrogen to carbon minimum in the formula is also verified. We conclude therefore, that the two analyzed samples are typical of the theoretical framework for these materials.

**Table 1:** Elemental analysis of the natural small molecule aliphatics, cholesterol and hexadecyl palmitate (the results shown in the table are expressed in atomic %)

Sample	C	H	N	S	O
Cholesterol (theoretical)	83.87	11.99	0.00	0.00	4.14
Cholesterol (found)	81.66	12.11	0.00	0.01	6.22
Hexadecyl palmitate (theoretical)	79.93	13.42	0.00	0.00	6.65
Hexadecyl Palmitate (found)	78.85	13.85	0.00	0.00	7.30

**Fig. 1:**
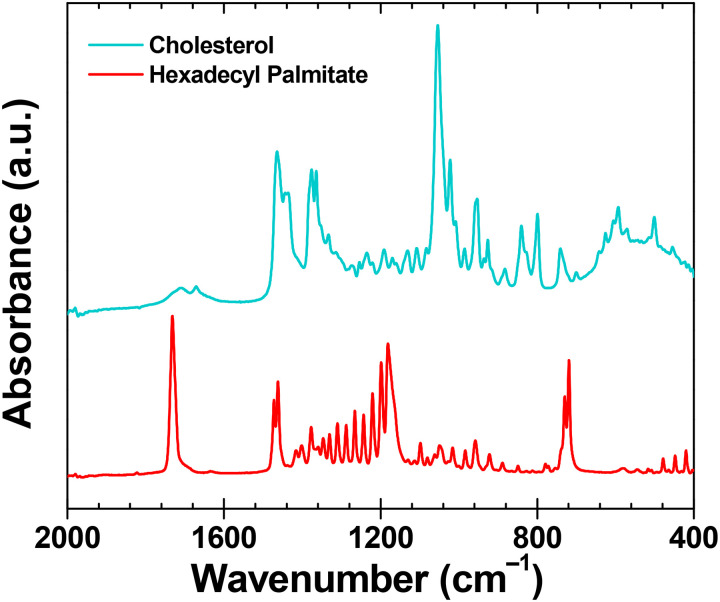
ATR-FTIR spectra of the analyzed small molecule aliphatics, cholesterol (cyan line) and hexadecyl palmitate (red line) in the spectral range 2000–400 cm^−1^.

The FTIR spectra confirm significant structural differences between the two small molecule aliphatics examined in this study. Fig. 1 highlights the spectral region containing the most critical features. The cholesterol spectrum in the fingerprint region reflects its more complex molecular structure, prominently featuring the intense C–O vibration of the alcohol group at 1054 cm^−1^. Additional characteristic bands include alkene functionalities (C=C stretching at 1450 cm^−1^; C=C ring bending, and C–H out-of-plane vibrations between 600–1000 cm^−1^) and vibrations from methylene and methyl groups (C–H bending at 1440 cm^−1^, and CH_3_ umbrella bending at 1375 cm^−1^). In contrast, the hexadecyl palmitate spectrum is dominated by absorptions indicative of the ester group (C=O stretching at 1730 cm^−1^), accompanied by characteristic vibrations typical of long-chain CH_2_ groups (doublet peaks at 1450 cm^−1^ for bending, and at 720 cm^−1^ for long-chain rocking). Additionally, the distinct splitting of vibrational bands (particularly in the fingerprint region for C–H and C–O vibrations) illustrates the strong tendency of hexadecyl palmitate to form highly ordered crystalline structures in its solid state and corroborate well our AFM-KPFM findings of HDP films that will be elaborated in the following. All observed spectral features by FTIR of cholesterol and hexadecyl palmitate align very well with the reported observations in the existing literature.^124,125^

**Fig. 2:**
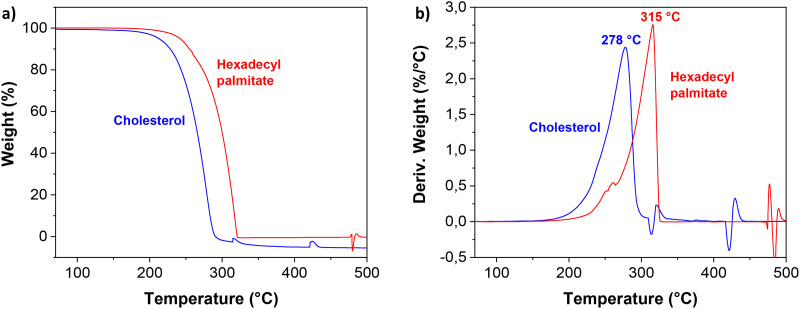
TGA analysis of the small molecule aliphatics displaying: (a) weight (%), and (b) derivative of weight loss.

The thermal stability of the cholesterol and hexadecyl palmitate samples was investigated by thermogravimetric analysis (TGA), the graphs being shown in Fig. 2. As can be seen from results, the two small molecule aliphatics showed similar thermal behavior to one another, yet slightly higher thermal stability was detected for hexadecyl palmitate as shown in Fig. 2. The exothermic reactions occur after heating the two materials above 200 °C. Their decomposition temperatures, *T*_d_, were found to be centered at 278 °C for cholesterol, and 315 °C for hexadecyl palmitate, respectively. From the comparative TGA (Fig. 2c), it can be observed that the decomposition process of the two materials showed only one main weight loss stage in the range of 200–330 °C, in accordance to other reported data.^126,127^ This main degradation zone (that accounted for more than 95% weight loss) was followed by two small second degradation zones for cholesterol, and one for hexadecyl palmitate, taking place at higher temperatures, where negligible mass loss occurred.

**Fig. 3:**
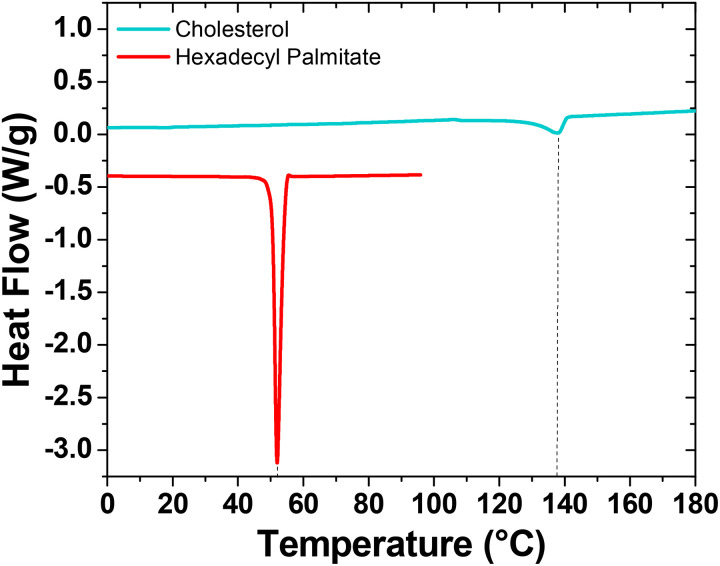
DSC thermograms from the second heating step for cholesterol and HDP. The two curves are offset for clarity.

Thermograms recorded during the second heating step of the differential scanning calorimetry (DSC) analysis are shown in Fig. 3. While thermogravimetric analysis confirms similar thermal stability against degradation for both aliphatics, calorimetric results highlight substantial differences in their melting behaviors. Hexadecyl palmitate exhibits a high degree of structural ordering, showing substantially more intensive melting endotherm at 52 °C (melting enthalpy 204 J g^−1^). In contrast, cholesterol demonstrates significantly lower crystallinity. However, the presence of an –OH group and the reduced flexibility resulting from its rigid cyclic structure notably elevate the melting temperature of cholesterol to 138 °C.

**Fig. 4:**
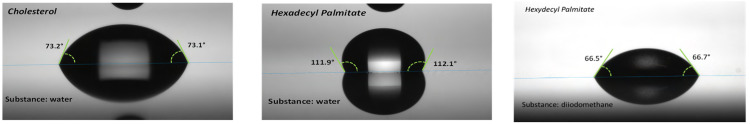
Contact angle with water droplet of cholesterol (left panel); contact angle with water and diiodomethane droplet of hexadecyl palmitate (center and right panels).

The contact angles with water and diiodomethane droplets of the two small molecule aliphatics are displayed in Fig. 4. Water and diiodomethane are the two classical probe liquids used in contact angle measurements. Water is polar, while diiodomethane is non-polar. By measuring the contact angles of both liquids and applying Young equation the polar and dispersive components of the surface energy of a solid can be determined. We run the measurement of cholesterol films several times, from freshly fabricated films and obtained similar results with the ones presented in Fig. 4, left panel. It is surprising and, in a way, counterintuitive to conclude that the cholesterol films cast from chloroform are in fact hydrophilic and not hydrophobic, although cholesterol is coined to a “fat-like” substance that is freely produced by many living species on Earth. The reason may be that its cholestane skeleton contains a 3β-hydroxy group that is expected to interact strongly with water, and has the only aliphatic section represented by its hydrocarbon chain connected on the C17 position to the central sterol nucleus (see Scheme 1). On the other hand, the hexadecyl palmitate film proved to be very hydrophobic, with a contact angle of ∼112° with water droplet and a contact angle of ∼67° with diiodomethane droplet, giving a total surface energy of approximately 24.7 mJ m^−2^, with no polar component.

The combined data of all our measurements with water and diiodomethane droplets for the two materials is summarized in Table 2. As Table 2 presents, the cholesterol film is not stable with diiodomethane, the respective droplet being immediately absorbed by the material, and because of this, we could not calculate the total energy of the cholesterol film. Moreover, the cholesterol film is not stable with water droplet either, its contact angle with water decreasing by 1*°* every 5 seconds; however, water is not instantaneously absorbed into the cholesterol film as is diiodomethane. For cholesterol films, we repeated the measurements on another instrument and obtained a value of 72.5*°* angle on several freshly fabricated samples *via* spin-coating, blade coating or simply *via* drop casting. In all the instances we indeed observed the same instability in time with the water droplet. Nevertheless, in all cases the measurements were performed in ambient air within a minute passed from the evaporation of the chloroform, and is possible that the film spontaneously oxidized and produced a layer of hydrophilic oxysterol that contributed to the recorded results.^128^ In addition, the cholesterol films aged for several weeks in ambient air provided a contact angle with water of ∼57.5*°*, and showed severe instability with water droplet, in line with the observations above, substantiating its high tendency for oxidation. The hexadecyl palmitate film on the other hand was stable when contacted with both liquids, with its total surface energy being equal to the dispersive component, 24.7 ± 0.4 mJ m^−2^.

**Table 2:** Overview of the contact angle measurement with water and diiodomethane droplets for cholesterol and hexadecyl palmitate; *measured 10 seconds after deposition. The water droplet is not stable on cholesterol, the contact angle decreases by 1° every 5 seconds; ** dioodomethane droplet dissolve cholesterol. *γ* represents the total energy, separable into a polar component, *γ*^P^, and a dispersive component, *γ*^D^

Material	Deposition Method	CA_Diiodomethane_ (°)	CA_H_2_O_ (°)	*γ* (mJ m^−2^)	*γ* ^D^ (mJ m^−2^)	*γ* ^P^ (mJ m^−2^)
Cholesterol	Spin coating	n/a**	72.8 ± 0.6*	n/a	n/a	n/a
Hexadecyl palmitate	Spin coating	66.8 ± 0.6	111.4 ± 0.9	24.7 ± 0.4	24.7 ± 0.4	0

**Fig. 5:**
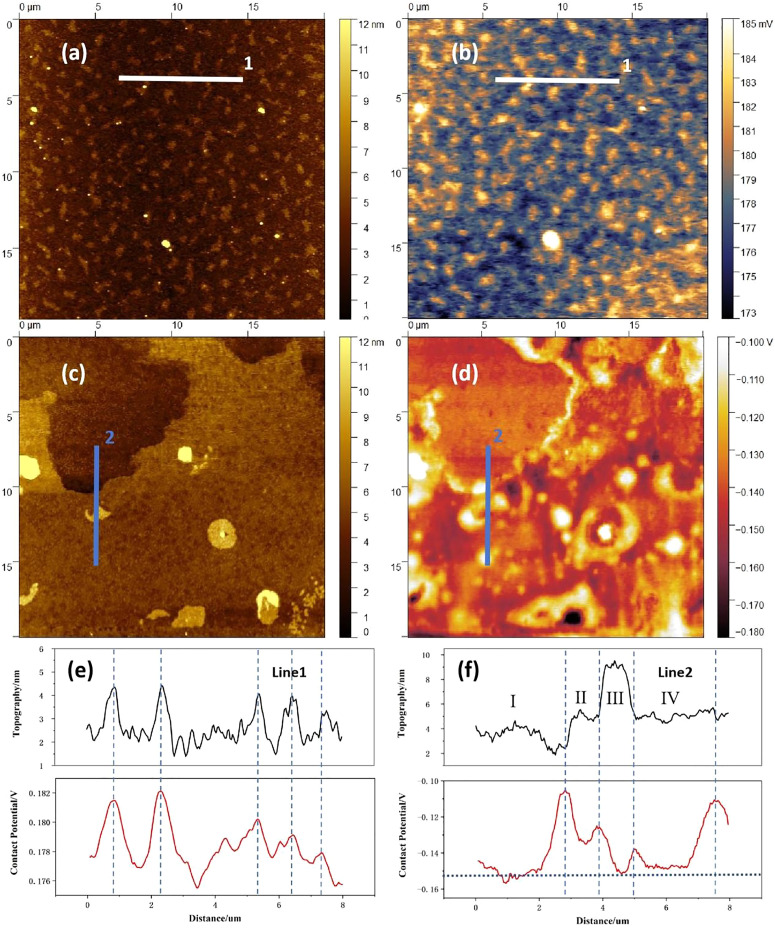
AFM-KPFM of investigated small molecule aliphatics: (a) cholesterol topography; (b) cholesterol contact potential difference; (c) hexadecyl palmitate topography; (d) hexadecyl palmitate contact potential difference; (e) cholesterol's topography (black) and contact potential (red) of Line 1; (f) HDP's topography (black) and contact potential (red) of Line 2. The dotted lines are displayed as visual guide of the peak positions.

The reactivity of the dielectric surface (its charge trapping action) at the corresponding interface towards the semiconductor material, can be assessed by the study of the contact potential difference (CPD). This interface trapping is especially noticeable in the case of inorganic dielectrics, and leads to the occurrence of both hysteresis and a decreased transistor current, *I*_ds_.^97^ The CPD is important to know also for organic dielectrics since, in addition to its traditional surface roughness (topography) analysis, it provides useful details regarding the surface quality of the dielectric films.^70,73^Fig. 5a and b presents the typical topography and contact potential difference (CPD) characteristics of the cholesterol film. The cholesterol surface exhibits numerous sub micrometer-sized islands with heights lower than 6 nm, demonstrating a root-mean-square (RMS) roughness of 1.7 ± 0.4 nm across five randomly measured surface areas. Notably, the CPD image correlates well with the topographic features, exhibiting a potential roughness of 2.9 ± 0.9 mV over the same measurement areas. Furthermore, it is noteworthy to mention that elevated regions (corresponding to cholesterol clustering) consistently display higher surface potential values, suggesting that CPD primarily arise from thickness variations. The surface morphology of HDP differs markedly from that of cholesterol (Fig. 5c). Three distinct morphological regions are observed: (i) low-lying plateaus (upper sample region, of ∼1–4 nm height, and width higher than 10 µm), (ii) intermediate terraces (dominant sample area, of ∼5–8 nm height), and (iii) protruding features (randomly distributed, of ∼8–60 nm height, and 1–2 µm width). The overall RMS of height is 1.5 ± 1.1 nm. While the HDP's CPD image exhibits some correlation with surface topography, a striking phenomenon emerges at interfacial regions: both the plateau-terrace boundaries and feature-terrace junctions consistently demonstrate elevated potentials. This interfacial enhancement explains why HDP exhibits substantially greater CPD roughness than cholesterol, *i.e.*, 13.9 ± 7.3 mV, despite their similar topographic roughness. The collection of results of AFM-KPFM investigations is displayed in Table 3). To analyze the mechanism of the interfacial enhancement, two-line sections were added for the two materials, *i.e.*, line 1 in the panels (a) and (b) and line 2 in the panels (c) and (d) of Fig. 5 respectively. It is worth to mention that line 1 and line 2 are both 200 pixels in length (∼8 µm) and 10 pixels in width (∼0.4 µm). Therefore, the signal of topography (black) and contact potential difference (red) are both average values of ten parallel lines. It can be clearly seen that these two molecules have different aggregation behavior and different contact potential range. It is worth pointing out that it is not the “up and down in the morphology profile” that directly leads to height variations of the CPD, but rather the chemical and electronic states of the material forming the analyzed surface and its fundamental properties, such as the type of aggregation and film growth, grain boundaries, dangling bonds, the presence of surface defects, induced stresses, *etc.*Fig. 5e shows that in the case of cholesterol, the peaks in the contact potential difference graph perfectly fit the respective peaks in the topography diagram. Therefore, it can be assumed that the density of defects on the surface of sub micrometer-sized islands is higher than that in other parts of the film. In the case of HDP (Fig. 5f), however, the peaks in the contact potential difference do not fit the peaks but the slopes of the left and right edges displayed in the topography graph. The contact potential of 4 terraces in Fig. 5f, especially the ones of the terraces I, III and IV (since the size of terrace II is very small, its signal might be submerged in two strong signals of the left and right slopes) are basically the same, even though their heights vary from 4 nm to 9 nm. Considering that there are a large number of chemical defects at the edge part, the local electrons are easily captured by these defects, forming short-range atomic dipoles.

**Table 3:** AFM-KPFM parameters of cholesterol and hexadecyl palmitate averaged over 6 measured surfaces

Material	RMS surface roughness (nm)	RMS contact potential difference (mV)
Cholesterol	1.7 ± 0.4	2.9 ± 0.9
Hexadecyl palmitate	1.5 ± 1.1	13.9 ± 7.3

The local contact potential can be defined as:

1 *Φ*_local_ = *E*_vac local_ − *E*_F_

where *Φ*_local_, *E*_vac local_ and *E*_F_, are the local work function, local vacuum energy level, and Femi energy level respectively. Since *E*_F_ must be maintained as a horizontal line, these short-range atomic dipoles present on the edge parts must cause *E*_vac local_ up-bending, and generate *Φ*_local_ (CPD) value larger than that of other parts of the sample. Therefore, the edge parts are not only physical boundaries, but represent also boundaries of electronic transport. Since the sub micrometer-sized islands can be considered as very small terraces combined with surrounding edge parts, their wide distribution could explain why the peak widths of the contact potential difference is wider than those of the topography for HDP. A similar observation of high potential difference at the edge of the steps compared to the terraces was reported by Y. Yamagishi *et al.*,^129^ on their KPFM analysis of C_8_-BTBT organic semiconductor, proving that crystallinity, layer by layer growth and packing structure play a crucial role for organic films.

**Fig. 6:**
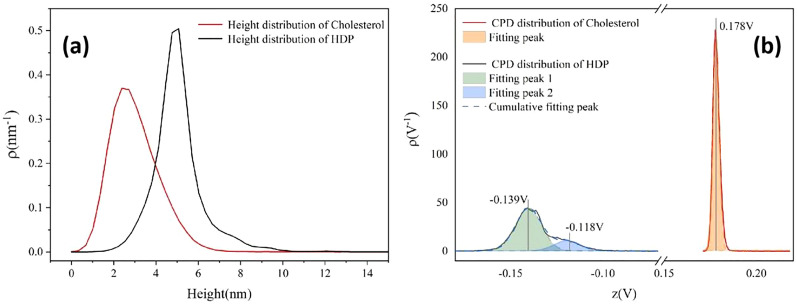
(a) Height distribution of cholesterol and hexadecyl palmitate, (b) CPD distribution of cholesterol and hexadecyl palmitate.

In order to further evaluate the aggregation behavior and electrical properties of the two substances, we generated Fig. 6, that shows the height and the CPD distributions for cholesterol and hexadecyl palmitate in the whole measuring area. The cholesterol samples exhibit congruent distribution profiles (red lines), with both parameters following normal distributions. The height distribution spans between 0 nm to 6 nm, consistent with the color bar in Fig. 5a, while the CPD distribution is sharply confined to 0.173 V to 0.185 V, reflecting its lower potential roughness. Although HDP's height distribution (*i.e.*, 2–8 nm range) resembles the one of cholesterol, its CPD characteristics differ substantially. The broader CPD distribution range in HDP, deconvolute into two distinct bands with their peaks centered at −0.139 V (the signal attributed to terraces) and −0.118 V (the signal attributed to the edges of the terraces) respectively, as elaborated during the analysis of Fig. 5f. Considering molecular structures of cholesterol and HDP, there are rigid steroidal rings and flexible alkyl chains, as well as hydrophilic hydroxyl groups in a cholesterol molecule, while there are only two completely symmetrical and flexible long alkyl chains in HDP. The hydroxyl (–OH) in cholesterol can form hydrogen bonds, which is a medium strength force with high directivity. It acts as a “positioning pin” to guide the arrangement of molecules in a specific way. At the same time, its huge rigid steroidal ring provides a strong and anisotropic van der Waals force, which promotes the orderly accumulation of molecules like a deck of playing cards. In contrast, HDP is an ester molecule (R–COO–R′), with a weekly polar ester group. It has no ability to form hydrogen bonds at all. Therefore, the only force that guides the interaction between HDP molecules is the van der Waals force between the long alkyl chains. This force has no directionality and is weaker than the van der Waals force between cholesterol rings. Therefore, during the film forming, cholesterol molecules self-assemble, forming a smectic-like structure, while HDP molecules arrange in a completely random physical entanglement, forming an amorphous structure. Consequently, the surface of cholesterol film is much smoother than that of HDP, consisting only in sub-micrometer-sized islands. In contrast, the HDP film contains step-like structures with their width exceeding 10 micrometers. In addition, the number of defects at the edge parts in cholesterol film are much less than that in HDP film. That is the reason why the distribution range of CPD signal of HDP is larger (−0.16 V to −0.10V) and deconvolute into two distinct bands (see Fig. 6b).

The dielectric capacitance is a material property that depends on the thickness (*d*), the measurement area of the material under investigation (*A*), as well as its relative permittivity (*ε*_r_):2
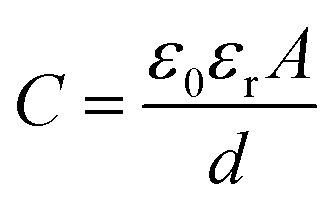
where *ε*_0_ is the vacuum permittivity, 8.854 × 10^−12^ Farads per meter (F m^−1^).

Relative permittivity, *ε*_r_, is usually symbolized as *ε*_r_ (*ω*), and is expressed by the formula:3
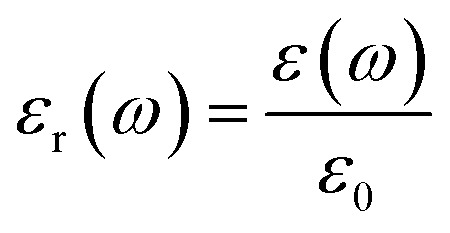
where *ε*(*ω*) is the complex (frequency-dependent) permittivity of the material. Stated otherwise, the relative permittivity is a complex, dimensionless number that can be written as:4
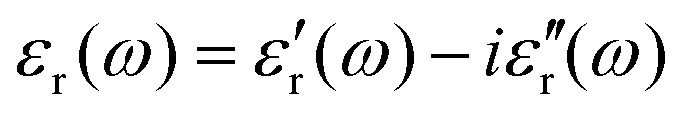


In general, the term “permittivity” is used to refer to only the real component 
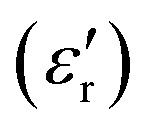
 of the complex-valued relative permittivity expressed in the eqn (4). By comparing their real *ε*′ and imaginary *ε*″ components (*i.e.*, their conductivity, *σ*), materials can be categorized based on their complex-valued permittivity, *ε*_r_(*ω*); thus, a perfect dielectric is a material with no conductivity at all (*σ* = 0), while a perfect conductor is a material an infinite conductivity (*σ* = ∞). The perfect dielectric (*i.e.*, in other words an ideal material with zero imaginary component 
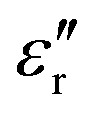
 of the complex permittivity) is also known as a lossless medium, or in other words, a medium without losses. In reality every material presents some sort of losses, since there is no such “perfect dielectric material”, and the cut-off is expressed by the ratio:5
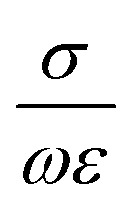


When the ratio expressed above is ≫1, the material is said to be a good conductor (*i.e.*, a lossy dielectric), and when the respective ratio is ≪1, the material is said to be a low loss dielectric, or in other words a very good dielectric. The electric loss tangent can be defined as:6
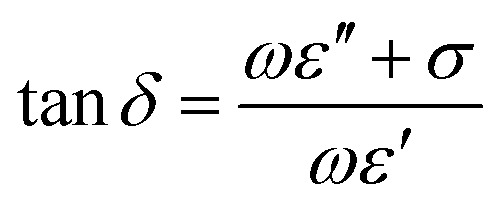
which can be simplified as:7
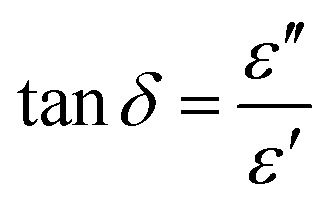
for a dielectric medium with low conductivity.

**Fig. 7:**
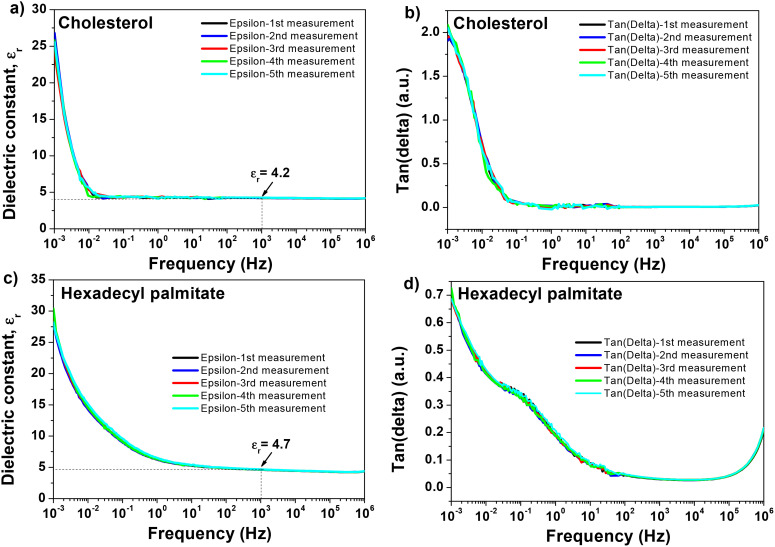
Five consecutive impedance spectroscopy of cholesterol, and hexadecyl palmitate in the frequency window from 1 MHz to 1 mHz. Each measurement duration was 18 hours. (a) dielectric constant of cholesterol measured on five consecutive scans; (b) the loss angle (tangent delta) for cholesterol measured on five consecutive scans. The sample thickness was measured as 127 nm; (c) dielectric constant of hexadecyl palmitate measured on five consecutive scans; (d) the loss angle (tangent delta) for hexadecyl palmitate measured on five consecutive scans. The sample thickness was measured as 95 nm.

The amplitude and time scale of charge density changes across the studied MIM sample are characterized by the behavior of the frequency-dependent *ε*_r_ and tan *δ*. Electronic polarization, ionic polarization, molecule orientation, chain relaxation, or polarization of free counterions, space charges (interfacial or condensed counterions), and electrode or electric double-layer (EDL) polarization are the main causes of these frequency-dependent oscillations.^130,131^ Impedance spectroscopy helps understanding interface processes, particularly those involving modifications to the system's mechanical, electrical, compositional, or crystallographic characteristics.^132^ It also helps understanding polarization and other changes in electrical characteristics by examining how they affect the system's electrical conductivity.^133,134^ The benefit of impedance spectroscopy is that it allows for the measurement of conductivity across a wide range of frequencies, making possible to learn about the conductive species and their routes in the analyzed material. In this work, we performed impedance spectroscopy on thin films of doctor bladed cholesterol and hexadecyl palmitate from chloroform solutions, by scanning the investigated metal–insulator–metal (MIM) structures in a wide range of frequencies, *i.e.*, from 1 MHz to 1 mHz. The result of one particular MIM structure investigation for each material is shown in Fig. 7, with all the measurements performed in ambient air. The typical thickness of the films of investigated materials processed from chloroform solution *via* blade-coating technique were ∼90–135 nm for hexadecyl palmitate and ∼100–150 nm for chloroform. The major drawback of the blade-coating deposition process from a highly volatile solvent as chloroform is that the remnant material at the interface between the blade and substrate spills back into the film and increases the thickness of the deposited film at the end-side position of the coated slide. Although this discrepancy is not very severe for the MIM structure, given the proximity of the MIM devices on the respective slide (see the photography of our slide design in the ref. 70), it translates, however, in a more significant variation of the transistors’ performance as it will be detailed in the following. In order to study the stability of the samples, we scanned each MIM sample of cholesterol and HDP five consecutive scans. The data presented in Fig. 7 shows a very robust resistance to the exposure to ambient air for both materials, with virtually no variation over 5 consecutive scans. It is worth mentioning that each individual measurement in the sequence (in the frequency window from 1 MHz to 1 mHz, having the fine point increment selected for the frequency step) lasted 18 hours. From the impedance measurements shown in Fig. 7, considering the thicknesses of the MIMs measured in the vicinity of the structure by profilometry, and the capacitance value measured at 1 kHz, we calculated the dielectric constant of the two materials. We obtained for hexadecyl palmitate a range of values for the dielectric constant between 4.2 and 4.8, and for cholesterol a range between 3.9 to 4.6, respectively. The particular samples shown in Fig. 7 have a dielectric constant of 4.2 in the case of cholesterol and 4.7 in the case of HDP. Interestingly, although used as received, without any purification other than a simple filtration of the precursor solution of the two investigated molecules, the cast film of hexadecyl palmitate seems to have a characteristic relaxation of the loss angle in the region that can be attributed to mobile ionic impurities (*i.e.*, a shoulder centered at ∼0.5 Hz, see Fig. 7d). It is however difficult to attribute this fact to the presence of a small hysteresis in OFETs with HDP, as the next section will demonstrate.

In contrast, the relaxation in the loss angle displayed by the cholesterol dielectric (see Fig. 7a) starts occurring at frequencies in the range of mHz or lower, and is not translated into a hysteresis in OFETs. This relaxation (*i.e.*, see the sharp increase and the recorded value of 2 for the loss angle of cholesterol dielectric at 1 mHz) most likely results from free charges and/or ions moving toward the electrode/sample interface under the influence of an electric field, which causes the formation of electric/ionic double layers in certain areas.^98^

**Fig. 8:**
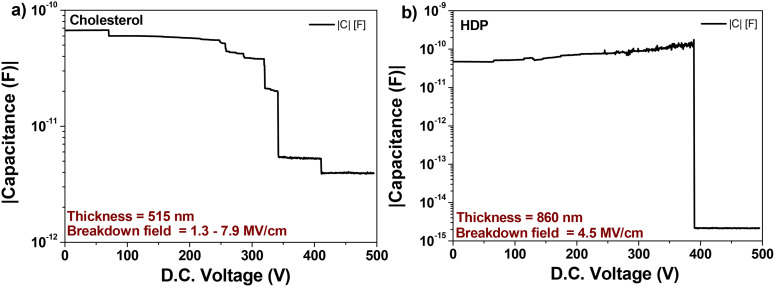
Breakdown field for (a) cholesterol, and (b) hexadecyl palmitate. The thickness of each sample is indicated as inset of the two graphs.

The breakdown field for the two materials is presented in Fig. 8a and b. Measured for samples with similar thicknesses to the one presented in Fig. 8, the two materials showed clear differences in their behavior with respect to breakdown field. The cholesterol samples broke in several stages, each being accompanied by a characteristic spark and a burn out of a small area of the two overlapping electrodes. The breakdown field for cholesterol is 1.3 MV cm^−1^ in the first breakdown stage, and 7.9 MV cm^−1^ in the last stage. In is worth mentioning that the cholesterol samples with thicknesses of the film higher than 750 nm could not be subjected to breakdown on our instrument that has a maximum range of 500 V applied D.C. voltage. In the same time, the HDP samples broke in one stage only (breakdown field of 4.5 MV cm^−1^), with the damage occurring in one event only and being distributed uniformly over the entire overlapped area of the top and bottom electrodes. Our attempt to perform the breakdown field test on thinner samples proved inconsistent results, possibly due to nonuniformity of the films and the difficulty to blade coat a uniform film on a mask size of 1.5 × 1.5 cm^2^. The values of the breakdown field recorded for the two analyzed small molecules is in between the one of low-*k* polymer dielectrics that lies between 1 and 2 MV cm^−1^ and synthetic polymer resins such as benzocyclobutene (BCB) that has a dielectric strength of 4.5 MV cm^−1^.^98^ These values are consistent with the trend observed for other investigated small molecules reported previously by our group (caffeine, theobromine and theophylline), also processed from highly volatile solvent like chloroform.^71^ However, it is known that the dielectric strength is dependent on the film thickness, expressed by the equation:

8 *V* = *At*^2/3^,

where *t* is the film thickness and *A* is a material constant. As a consequence, the dielectric strength increases as the thickness of the dielectric film decreases.^135^ Because the breakdown field values shown in this work only represent a narrow sample thickness range and are highly specific to the thickness of the dielectric films utilized, they might need to be reexamined in a more methodical examination.

**Fig. 9:**
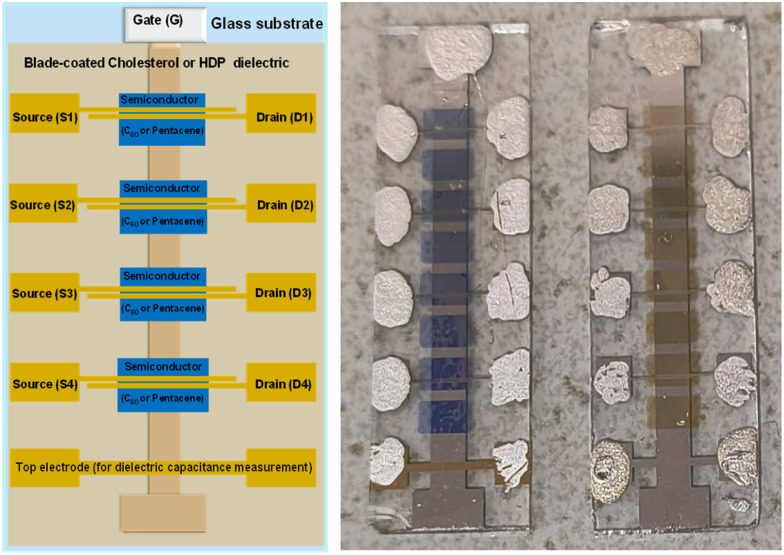
(Left panel) OFET design utilized in this work; (right panel) Photograph of fabricated devices with pentacene (left slide) and C_60_ (right slide) organic semiconductors. The silver paste was added on the measurement pads in order to improve the contact between the sharp needles of the probe station and the measured contact terminals.

The schematic utilized for the fabrication of the OFETs is presented in Fig. 9. We fabricated two batches of 6 slides each for each of the two dielectrics, both with C_60_ and pentacene semiconductors, making in total a pool of 48 OFETs for each combination of dielectric and semiconductor. During the measurement of the devices, we noticed that the OFETs marked with the number 4 on the slide (see Fig. 9) functioned at a slightly higher voltage that the OFETs in position number 1, which was the starting point of the blade coating. We measured the thickness of the film in various positions of the slide and concluded that it is significantly higher at the end of it, which represents the finish point of the blade coating. We believe that the dexterity of the of operator to remove the excess material played a major role in obtaining reproducible results with organic materials processed by blade coating from highly volatile solvents like chloroform, since the solvent both evaporates very fast and has a tendency to spill back into the already deposited film, contributing to its increased thickness at the end of the slide.

**Fig. 10:**
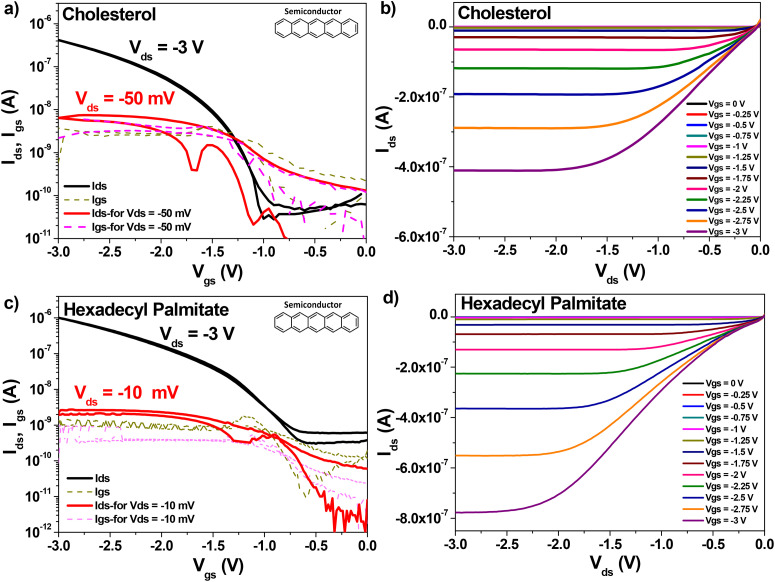
Transfer and Output OFET measurements with pentacene as organic semiconductor. (a) and (b) cholesterol dielectric on plain aluminum gate electrode: specific capacitance, *C*_0d_ = 73.1 nF cm^−2^, field effect mobility *µ* = 4.2 × 10^−2^ cm^2^ V^−1^ s^−1^, subthreshold swing, *S*_SW_ = 95 mV dec^−1^. (c) and (d) Hexadecyl palmitate on plain aluminum gate electrode, specific capacitance, *C*_0d_ = 73.1 nF cm^−2^ field effect mobility *µ* = 5.2 × 10^−2^ cm^2^ Vs^−1^, subthreshold swing, *S*_SW_ = 335 mV dec^−1^.

**Fig. 11:**
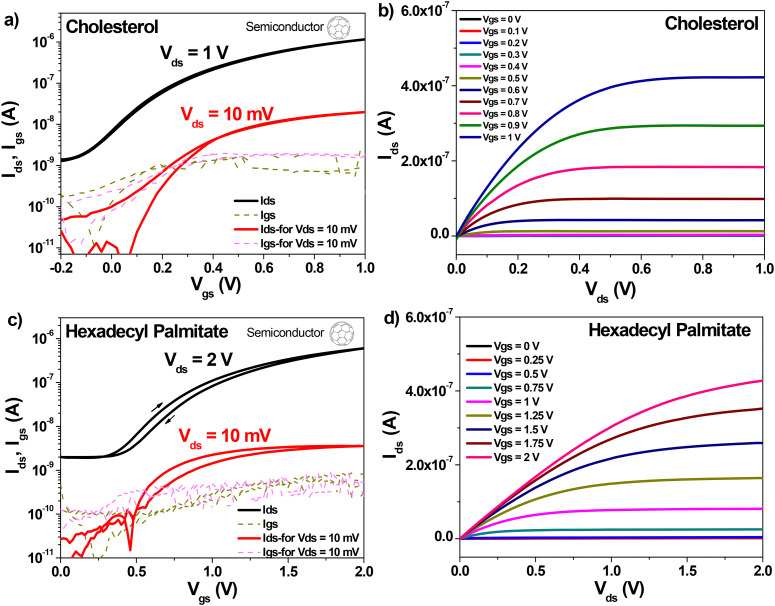
Transfer and Output OFET measurements with fullerene, C_60_ as organic semiconductor. (a) and (b) cholesterol dielectric on plain aluminum gate electrode: specific capacitance, *C*_0d_ = 68.2 nF cm^−2^, field effect mobility *µ* = 0.48 cm^2^ V^−1^ s^−1^, subthreshold swing, *S*_SW_ = 245 mV dec^−1^. (c) and (d) Hexadecyl palmitate on plain aluminum gate electrode, specific capacitance, *C*_0d_ = 97.9 nF cm^−2^ field effect mobility *µ* = 6.2 × 10^−2^ cm^2^ V^−1^ s^−1^, subthreshold swing, *S*_SW_ = 320 mV dec^−1^.

Because of this uncertainty of the dielectric thickness, we did not use the values of the specific capacitance measured with the aid of the continuous electrode (see Fig. 9), since the respective value was not representative to the capacitances of individual OFETs on the slide. We fabricated instead MIM devices with the top electrode deposited in the position of the four OFETs on the slide and used the respective capacitance for the semiconductor field effect mobility calculation. The transfer and output OFET measurements with the two dielectric materials are presented in Fig. 10, when interfaced with the p-type semiconductor, pentacene, and in Fig. 11, when interfaced with the n-type semiconductor fullerene, C_60_, respectively. Although we measured devices at higher operating voltages, *i.e.*, up to 5 V for pentacene semiconductor interfaced with the two small molecules dielectrics, and up to 4 V for the case of C_60_, we present in the two figures example of devices for each combination of dielectric and semiconductor.

The OFET parameters are inserted in the panel of transfer characteristics for each OFET combination and are summarized together in Table 4. In addition to their low voltage operation window of 1–3 V, the fabricated OFETs can be run with a small applied drain-source voltage as low as 10 mV, and not higher than 50 mV in most of the cases. Inspecting the two figures, one observes that the two dielectric materials generated similar results with fullerene, C_60_ and pentacene respectively, with mobilities in the range of 10^−2^ cm^2^ V^−1^ s^−1^, ON–OFF ratio in excess of 1000, and similar subthreshold swing, as the data compiled in Table 4 confirms. Also, it is noticeable that the interface of the two dielectrics towards pentacene generates a much lower OFF level of the OFET devices. In addition, the interface of the two small molecules dielectrics towards C_60_ gives rise to a small hysteresis in the transfer characteristics, more visible in the case of HDP, while there is also a variation of the threshold voltage towards negative values in the case of cholesterol, possibly due to the difference in morphology of the C_60_ film. Nevertheless, this small but negative threshold voltage for the OFET devices with cholesterol and C_60_ was not universally present, some samples displayed a small and positive threshold value of *V*_th_ in their transfer characteristics.

**Table 4:** OFET parameters of natural small molecule aliphatics on aluminum gate, with pentacene and C_60_ semiconductors. *The record mobility measured on the cholesterol + C_60_ device was an exception, the values of the field effect mobility were no greater than 0.1 cm^2^ Vs^−1^ for the rest of the OFETs in the fabrication pool

Aliphatic molecule	OFET parameters									
	with Pentacene					with C_60_				
	*C*_0d_ (nF cm^−2^)	*V*_th_ (V)	*I*_ON_/*I*_OFF_	*µ* (cm^2^ V^−1^ s^−1^)	*S*_SW_ (mV dec^−1^	*C*_0d_ (nF cm^−2^)	*V*_th_ (V)	*I*_ON_/*I*_OFF_	*µ* (cm^2^ V^−1^ s^−1^)	*S*_SW_ (mV dec^−1^)
Cholesterol	73.1	−1.25	3.35 × 10^3^	4.2 × 10^−2^	95	68.2	−0.1	920	0.48*	245
Hexadecyl palmitate	101.1	−1.15	3.21 × 10^3^	5.2 × 10^−2^	335	97.9	0.3	302	6.2 × 10^−2^	320

## 4. Conclusions

In this report, we complement the contributions of the scientific community towards the field of sustainable electronics by demonstrating that two small molecules aliphatics, cholesterol and hexadecyl palmitate are excellent dielectrics to consider for the fabrication of environmentally friendly electronics. Different than our recent work on natural dielectrics,^65,69,70,73^ this study focuses on two well defined materials with a unique composition, *i.e.*, cholesterol and hexadecyl palmitate, leaving no room for the assertion that a difference in material origin and/or its complex constituency may generate different results. We demonstrated that these ubiquitous small molecules can be employed as dielectrics in the fabrication of organic field effect transistors operating at low voltages. Contrary to the common belief, cholesterol does not behave as a hydrophobic material when exposed to ambient atmosphere, and can be processed from a variety of solvents, many of them considered “green”. We processed it from chloroform in order to have a direct measure for comparison with hexadecyl palmitate that is not soluble in cold, “green” solvents except oils (*n.b.*, HDP is in fact soluble in boiling anhydrous ethanol and even in dichloromethane at room temperature, the latter being a solvent used in food industry for coffee, cocoa, and tea decaffeination, as well as for various flavors extraction).^136^ Both small molecule aliphatics allow the fabrication of OFET devices virtually hysteresis-free, in both transfer and output characteristics. The two small molecule dielectric materials investigated here compare favorably with a plethora of other small molecule or polymer dielectrics in their performance when employed as insulating layer in OFETs. Among the exhaustive list of polymer dielectrics reported elsewhere, the PMMA^91–94^ and SU8^96^ stand apart, since their dielectric constant is similar to the one measured in this study for cholesterol and HDP. With this respect the main OFET parameters, *i.e.*, field effect mobilities recorded, the subthreshold swing and on–off ratio measured for the two materials investigated here are similar with the reported results in the above references. Different than the literature reports however, the OFET devices fabricated in this work can be operated reliably in the 1 V operation window of the gate voltage, with applied drain-source voltages as low as 10 mV. Although not as versatile as shellac and silk, *i.e.*, two other natural biopolymer materials that are also solution processible and can be employed both as substrates and dielectrics for organic electronics, the two small molecules investigated here compare successfully to other materials of natural origin exemplified above in terms of dielectric strength, and low voltage afforded in the operation of the organic field effect transistors. In line with our recent work on natural dielectrics,^30,57,65,66,69–71,73,74^ it seems reasonable to assume that mother nature still has plenty to offer on the side of materials that are biocompatible, biodegradable, and do not pose any threat to life and environment; and that these two small molecules are only two examples of many more natural compounds that can be implemented successfully in electronics fabrication.

## Conflicts of interest

The authors declare no conflicts of interest.

## Data Availability

Data are available upon request from the authors.
